# Exploration of the Principal Component Analysis (PCA) Approach in Synthesizing the Diet Quality of the Malaysian Population

**DOI:** 10.3390/nu13010070

**Published:** 2020-12-28

**Authors:** Asma’ Ali, Barrie M. Margetts, Ahmad Ali Zainuddin

**Affiliations:** 1 School of Food Science & Technology, Universiti Malaysia Terengganu, Kuala Nerus 21030, Malaysia; 2 Faculty of Medicine, University of Southampton, Building 85, Life Sciences Building, Highfield Campus, Southampton SO171BJ, UK; B.M.Margetts@soton.ac.uk; 3 Institute for Public Health (IPH), National Institute of Health, Ministry of Health Malaysia, Seksyen 13 Setia Alam, Shah Alam 40170, Malaysia; ahmadali@moh.gov.my

**Keywords:** principal component analysis, diet quality, traditional diet, Malaysian population

## Abstract

(1) Background: One of the most main dietary assessments is through a posteriori application. Although extensive research has incorporated dietary assessment of a population through a posteriori application, this study is the first to examine the Malaysian population and use an a posteriori method and principal component analysis (PCA) to assess the dietary patterns of the Malaysian population. The correlation between all dietary patterns derived via PCA and selected nutrient intake were determined in this sample of study; (2) Methods: A total of 3063 respondents (18 to 59 years old) covering Peninsular Malaysia, Sabah, and Sarawak, participated in this study. PCA was applied on the food frequency questionnaire collected from the respondents, and descriptive statistics and PCA were performed using SPSS version 21; (3) Results: Six patterns were identified: “traditional”, “ prudent”, “ modern”, “western”, “Chinese”, and “combination” diets. All together, these six patterns were able to explain 45.9% of the total variability. Few components derived from the factor loadings showed positive association with several nutrient markers. The traditional dietary pattern showed a moderate, positive correlation with total protein and total sugar intake, there was a significant moderate correlation between the prudent dietary pattern and dietary fibre, and there was a moderate positive association between the Chinese dietary pattern and total energy; and (4) Conclusions: The exploration of the PCA approach above may provide justification for assessment of dietary patterns rather than reliance on single nutrients or foods to identify potential connections to overall nutritional wellbeing as well as to explore the diet–disease relationship. However, study of pattern analysis must be conducted among the Malaysian population to produce validity and reproducibility for this dietary approach in light of the numerous methodological issues that arise when performing PCA.

## 1. Introduction

In September 2011, the United Nations General Assembly (UNGA) passed a resolution on the Prevention and Control of Non-communicable diseases (NCDs) that clearly highlighted the importance of poor nutrition and early undernutrition as major causes of NCDs in lower and middle-income countries (LMICs). This resolution underlined the need for action to improve diet and physical activity patterns worldwide to curb the rise in obesity and other chronic, diet-related diseases. Overnutrition has dramatically expanded in Malaysia, and although the rate of undernutrition has been reduced, hunger remains a significant problem among isolated groups. As a result, the particular challenges for Malaysia and similar, newly emerging economic powers are twofold: how to ensure that both over- and undernutrition are appropriately addressed, and how to ensure that policies aimed at reducing undernutrition do not conversely spur overnutrition. The UNGA declaration highlighted key actions that member states (i.e., countries such as Malaysia) needed to take to prevent and control the spread of NCDs. These actions included changes in dietary patterns to increase consumption of fruits and vegetables while reducing intakes of fats, salt, and sugars. For Malaysia to achieve these changes, the first step is to identify the Malaysian population’s patterns of food consumption.

In the latter half of the 20th century, researchers started to study dietary patterns rather than the consumption of individual nutrients or foods [1] because people do not eat only certain nutrients or foods but rather a complex mixture of foods [2]. This dietary pattern approach was initially suggested during the White House Conference on Food, Nutrition and Health in 1969 [3]. However, it was not until 1981 that the US made its first attempt at applying the available data in studying the link between dietary patterns and nutritional status among the US population [3,4]. This innovative method explored diet and health in a way that transcended the traditional analyses used in the nutritional epidemiology of evaluating the effect of single foods or nutrients on the risk of developing various chronic diseases [5,6,7]. Kant (2004), Arvaniti and Panagiotakos (2008), and Wirt and Collins (2009), in their reviews of the literature on dietary patterns [6,8,9], demonstrated that studies had utilised at least one of two methods to determine dietary patterns in nutritional epidemiology: a priori and a posteriori methods. The a priori method is based on diet indices or scores that assess compliance with prevailing dietary guidelines, while a posteriori is a data-driven method that applies factor or cluster analysis to derive dietary patterns. 

Principal component analysis (PCA) is the most commonly used statistical technique in the a posteriori method. PCA deducts dietary variables through aggregation and produces a unique factor solution. These factor solutions were selected based on certain cut-off points of the eigenvalue, and the remaining factor solution will usually be concluded by naming of the factors. Many studies have explored PCA in determining the dietary patterns of a population [10,11]. Hearty and Gibney (2009) determined the dietary patterns of 1379 Irish adults, aged 18–64, involved in the North/South Ireland Food Consumption Survey 1997–1999, and they found that PCA approach resulted in “Traditional Irish”, “Healthy” and “Unhealthy” dietary patterns [12]. In another major study, Cunha et al. (2010) analysed three statistical methods in assessing the dietary patterns of 1009 adults, aged 20–65, living in Greater Rio de Janeiro, Brazil [13]; this cross-sectional study applied component analysis, cluster analysis, and reduced rank regression (RRR) in identifying dietary patterns among the subjects. The results proved that all three methods resulted in similar dietary patterns, where two distinct dietary patterns were revealed: “mixed” dietary patterns characterised by cereals, leafy greens, vegetables, roots, meat, eggs, sausage, and caffeinated beverages, and traditional dietary patterns consisting of rice, beans, and bread. The component analysis also yielded a western dietary pattern characterised by fast food, soft drinks, juice, milk and dairy, sweets, cakes, and cookies. 

Although there appears to be significant impact when an a posteriori application is utilised when assessing dietary patterns, this method has received little use in Malaysian studies. Therefore, this study will assess the dietary patterns of the Malaysian population using the a posteriori method, and PCA was selected to represent the method. By the end of this study, our objectives are as follows:

1. To identify the type of dietary patterns derived by the PCA approach in this study sample.
2. To determine the correlation among all dietary patterns derived by PCA and selected nutrient intake in this study sample.

## 2. Materials and Methods 

### 2.1. Study Design and Respondents

This study utilised data from the Malaysian Adult Nutrition Survey (MANS), the first Malaysian national nutrition survey, which was conducted from 2002 to 2003; covered Peninsular Malaysia, Sabah, and Sarawak; and was funded by Malaysia Ministry of Health. MANS was fully coordinated by the Nutrition Section of the Family Health Development Division under the Ministry of Health Malaysia; this division had assembled a technical committee that was responsible for developing the survey design and the survey questionnaire, monitoring the quality of the survey data, analysing the data, and preparing reports. MANS was conducted to determine the nutritional status and food consumption of Malaysian adults, and a total of 3063 respondents, aged 18 to 59 years old, participated in this study. Informed consent was acquired from respondents prior to their involvement in the survey. The data collection ran from October 2002 to July 2003 for Peninsular Malaysia and from January to December 2003 for Sabah and Sarawak. The custodianship of the MANS data was made the responsibility of the Institute of Public Health, Malaysia (IPH) and approval of data usage was obtained from the Director General of IPH. This research and the study protocol were approved by the Medical Research and Ethics Committee, Ministry of Health Malaysia, NMRR ID (NMRR-12-815-13100).

### 2.2. Sampling Frame

MANS analysed both urban and rural areas in western and eastern Malaysia, and only households in the private living quarters (LQs) were included. The study’s designers removed institutional households such as hostels, hotels, hospitals, and prisons, which accounted for just 1% of all households at the time. The sample frame was used for the 2000 Population and Housing Census Enumeration Blocks (EBs), the extent of which the National Household Sampling Frame Department of Statistics determined. The EBs in the sample frame are grouped according to the 2000 Population and Housing Census by urban and rural areas. The first stage of the sample unit is the EB, and the LQ in the same EB is the sample unit in stage two. EBs have been chosen using the “size-probability” form; EBs with a larger LQ size are therefore more likely to be chosen. The LQ in the selected EBs is the second step of the sample unit.

### 2.3. Survey Questionnaire 

The questionnaire consisted of five sections: socio-demography; 24-h diet recall and meal pattern; habitual physical activity and 24-h physical activity recall; anthropometry; frequency of food intake (via the food frequency questionnaire, or FFQ); and frequency of supplement intake. However, only socio-demography, anthropometry, and frequency of intake were selected because of the contribution of their data in fulfilling the requirement for analysis of this study. Other parts of the questionnaire have been explained elsewhere [14,15,16,17]. MANS used the semi-quantitative FFQ, which featured a list of 126 food items categorised into 13 food groups. There were a total of four main columns in the FFQ; the first column contained a list of food items, and the second column showed the frequency intake by day, week, month, and year—or not eaten at all. The frequency of intake was based on the habitual food intake during the past year. The third column described the serving size of each food item, while the fourth column described the number of servings consumed each time the food was eaten. The 13 food groups were cereals and cereal products (17 food items), meat and meat products (12 food items), fish and seafood (12 food items), eggs (4 food items), legumes and products (4 food items), milk and milk products (6 food items), vegetables (10 food items), fruits (20 food items), beverages (11 food items), alcoholic beverages (5 food items), confectioneries (8 food items), spreads (6 food items), and condiments/miscellaneous (11 food items); additional questions addressed the use of sugar, cooking oil, and salt by household members per month.

### 2.4. Dietary Intake

Dietary intake includes findings of the energy, macronutrient intake, and micronutrient intake of the respondents. The Nutritionist Pro^TM^ Diet analysis software was used to determine the respondents’ energy and nutrient intake levels. For the measurement of nutrient intake among respondents, food databases such as the Nutrient Composition of Malaysian Food and the USDA’s standard reference data were used. The calculation for converting FFQ data to daily energy and other nutrients is based on the conversation factor used to estimate food intake based on frequency of intake (cf. Wessex Institute of Public Health) [18]: amount of food consumed per day (g) = conversion factor (y) × type of serving size x total no. of serving size × weight of food in one serving. The energy, macronutrient intake, and micronutrient intake of the respondents were compared with Malaysian Recommended Nutrient Intake (RNI). The RNI is the daily intake which meets the nutrient requirements of almost all (97.5%) apparently healthy individuals. 

### 2.5. Food Groups 

The semi-quantitative FFQ included in MANS consists of 126 food items and contains a large number of variables to run the PCA. To run a PCA, the food data must be aggregated into groups [19] to ease the interpretation of factor components [12]. Therefore, the 126 food items were grouped into 17 food item groups, as shown in the Appendix A Table A1 and Table A2, according to similarity of nutritional characteristics [13,20], common classification [21] and similarity to MANS food groupings [22]. This method of food aggregation follows other studies in preparing food data for PCA analysis [5,23,24,25,26,27]. The analysis was based on grams per day weightage, a standard found in several studies [12,21]. No significant difference was found among weightage, adjusted energy, and percentage of energy contribution in determining factor loadings of PCA [28]. Therefore, grams per day has been selected as the weight for this PCA analysis. 

### 2.6. Identification of Dietary Patterns

PCA was applied to the 17 food item groups. Prior to performing PCA, the suitability of data for factor analysis was assessed. The Kaiser–Meyer–Olkin test, a measure of sampling adequacy, was applied to examine whether the variables are adequate for correlation [29]. Bartlett’s test of sphericity, a test to observe a relationship between variables, was also conducted prior to PCA analysis. PC factors were retained using the Kaiser criterion to use all eigenvalues greater than 1.0. PCA with a Varimax rotation has been selected, and the rotated component matrix will provide a clearer picture of factor loadings onto the identification of the dietary patterns. Variables with high loadings (≥0.2) have been selected for the rotated component matrix to ease the interpretation. Items that load <0.2 were excluded from a factor because less than 9% of that item’s variable is shared with that item’s factor [30]. Several studies on dietary pattern analysis using a factor analysis approach had considered absolute factor loadings >0.2 as having significance as a contribution to dietary pattern analysis [31]. To confirm the retained factors further, a scree plot was also used.

### 2.7. Statistical Analysis

All analyses were conducted using SPSS version 21 (IBM, Armonk, NY, USA). Preliminary analyses were implemented to avoid violation of the assumptions of normality, linearity, and homoscedasticity. A one-way between-group analysis of variance (ANOVA) was conducted to explore the differences between nutrient intakes by socio-demographic characteristics. Scores for all respondents on each identified pattern were generated in determining association between body mass index (BMI) and other nutritional profiles (e.g., fat, added sugar, saturated fat, salt, and dietary fibre). The Pearson product-moment correlation coefficient was employed to measure the association between BMI and the estimated factor scores. A two-sided *p*-value of <0.05 was considered significant.

## 3. Results

### 3.1. Sociodemographic Background

The socio-demographic background of the respondents is presented in Table 1. The national survey covered all states of Malaysia, including Sabah, and Sarawak, for a total of 3063 respondents. Almost 80% of the respondents came from Peninsular Malaysia, while the remaining were from Sabah and Sarawak. There was only a slight difference between the percentage of respondents from urban areas (52%) and those from rural areas (48%) and also between male (49%) and female respondents (51%). The urban population rose from 62.0% in 2000 to 62.5% in 2003 during the review period of Malaysia Eight Plan, 2001–2003, while the rural population declined from 38.0% in 2000 to 37.0% in 2003; this differed slightly from the proportion obtained via MANS. Despite the differences, the share of urban respondents is still greater than that of their rural counterparts due to increased migration, growth in new urban areas and the extension of administrative urban boundaries [32]. Of the total respondents, 52% were Malay, 24% were Chinese and 8% were Indian; the remaining 16% were Bumiputra Sabah and Sarawak as well as indigenous people. Most respondents were married (68%) and had a mean average age of 34.5 ± 11.1 years. Most had completed upper secondary school (35%) or at least lower secondary school (21%), while few had completed tertiary-level education (14.2%). A large percentage of the respondents were employed (64%). The mid-term review of the Eighth Malaysia Plan showed that the working age group (ages 15–64) constituted 62.7% of the Malaysian population in 2003, those below the age of 15 constituted 33.2%, and those aged 65 and above constituted 4.1%. MANS, in its assessment of working-age respondents, therefore reflected the Eight Malaysia Plan review [32]. The mean average household income for respondents was MYR 1992.61, roughly GBP 385 (GBP 1 = MYR 5.15), with approximately 55% of the population reporting a household income less than MYR 1500. The mean household income matched that observed in a study conducted by Haron et al. (2005) [33], who examined income levels in the state of Selangor, the most developed and urbanised state in Malaysia, and found that the average monthly household income was MYR 1730.11. Most surprising, both monthly household incomes were lower than the national average income of MYR 2472 [32]; the low household income was parallel with the educational attainment of the respondents, the majority of whom had completed the upper secondary level. BMI was calculated for all of the respondents according to the WHO’s (1998) classification scheme, and the mean average BMI for all the respondents was 23.5 ± 4.3 SD.

### 3.2. Dietary Intake of Respondents

The Malaysian Ministry of Health has adopted RNIs as the nomenclature for reference to dietary recommendations. The total mean energy intake of all respondents was 2293 kcal ± 657 SD. RNIs for energy and other nutrients are age and gender dependent, and therefore we categorise men and women into age groups to investigate the mean percentage of RNI achieved for each age group, as shown in Table 2 and Table 3. Total energy intake for men and women was 2472 kcal ± 411 SD and 2125 kcal ± 374 SD, respectively. RNI for total energy intake for the Malaysian population is around 2440 kcal to 2460 kcal for men and 2000 kcal to 2180 kcal for women. A significant difference in mean energy intake was found between men and women—*t* (3009) = 24.5, *p* < 0.001—with men having a greater energy intake than women had. Table 4 shows the distribution of total energy intake and other nutrient intake among the Malay, Chinese, and Indian populations. The mean average of total energy intake was higher among Malays, followed by Chinese people, with Indians demonstrating the lowest average. However, there was no significant difference in the total energy intake among the Malay, Chinese, and Indian populations. A one-way inter-group analysis of variance was conducted to explore the differences in macronutrient intake among Malay, Chinese, and Indian populations, and statistically significant differences at *p* < 0.05 were found in protein and fat intake. Hence, a two-way inter-group analysis of variance was performed to explore the impact of gender on protein and fat intake among the Malay, Chinese, and Indian populations. The interaction effect between gender and ethnicity group was not statistically significant for protein intake (gender*ethnicity = 0.501), which indicates that there is no significant difference in the effect of gender on protein intake for the Malay, Chinese, and Indian populations; F (3, 3055) = 0.79, *p* = 0.50. There was a statistically significant main effect for ethnicity: F (3, 3055) = 21.3, *p* < 0.001; however, the effect size was small (partial η^2^ = 0.02). Next, two-way inter-group analysis of variance was performed on fat intake among the Malay, Chinese, and Indian populations to assess the impact of gender. The interaction effect between gender and ethnicity group was not statistically significant for fat intake (gender*ethnicity = 0.233). However, there was a statistically significant main effect of fat intake for ethnicity—F (3, 3055) = 4.075, *p* < 0.0—where the effect size was extremely small (partial η^2^ = 0.004).

### 3.3. Dietary Patterns Derived by PCA Approach

With the Kaiser criterion, there are six components (factors) of an eigenvalue greater than 1.0. After six factors, the scree plot also demonstrates a noticeable break. Factor 1 accounts for 9.862% of the variance. The rotated component matrix details the factor loadings onto the six components. Table 5 provides the loadings of the variables onto the factors. The rotated component matrix shows variables that are of paramount importance to Factor 1. Accordingly, six components—”traditional”, “prudent”, “modern”, “western”, “Chinese”, and “combination” diets—were chosen to best describe the dietary patterns of the respondents, explaining 45.9% of the variance. An earlier study conducted in Malaysia revealed four patterns within the Malaysian dietary pattern: modern, prudent, traditional, and combination, explaining 69.4% of the total variance [34]. Following rotation, six dietary patterns were derived to describe most clearly the dietary patterns of the respondents in this sample of study. The names addressed to each pattern were based on an understanding of the content of the variables (based on past research). Factor 1 was the most dominant food pattern within the population and explained 9.9% of the variance intake, whereas each of the remaining five factors explained between 9.5% (Factor 2) and 5.9% (Factor 6) of the variance. 

The first pattern, Factor 1, with high loadings for flavourings, fish and seafood, confectioneries, eggs, and non-alcoholic beverages was labelled the traditional dietary pattern. This traditional dietary pattern was also associated with lower intake of milk, other types of meat, cereals, and cereal products, and this pattern was labelled traditional because of the rich consumption of flavourings in Malaysian dishes, which include sugar, honey, shrimp sauce, anchovy sauce, shrimp cencalok, thick soy sauce, light soy sauce, ketchup sauce, oyster sauce, and fish sauce. These flavourings are added frequently during food preparation in traditional Malaysian cuisine or are served as side dishes. The most popular dish in Malaysia is nasi lemak (coconut rice), a staple of breakfast and dinner alike in which rice is cooked in a mixture of coconut milk and water. A gravy made with shrimp sauce along with chillies, sugar, and anchovies is then added to the rice. Even though the quantity of this gravy is ostensibly negligible, if it is consumed regularly, the shrimp sauce nevertheless yields an impact on dietary patterns. Other commonly consumed traditional dishes are fried rice and fried noodles; preparation of these foods involves the use of thick soy sauce, light soy sauce, and ketchup sauce. Another popular traditional dish, especially among both the Malay and Chinese populations, is chicken rice, in which the chicken is prepared through marination with soy sauce and honey. Fish and seafood, along with confectioneries, loaded moderately on Factor 1. Fish is the predominant source of protein in Southeast Asia [35]; Chinese Malaysians tend to consume fish such as Spanish mackerel, silver pomfret, and anchovies while Malays often opt for Indian mackerel, black pomfret, hardtail pomfret, sardines, and anchovies [36]. Sheng et al. (2008) calculated that fish consumption in Malaysia was 56.39 kg/person/year in the year 2003, which accounted for 12.4% of the total food intake per capita [37]. An additional important score on Factor 1 entailed confectioneries, which include local delicacies, cake and biscuits, and have served as traditional tea-time dishes for the Malaysian population. There are a wide range of choices of local delicacies, which may be prepared in the home or available from almost every food vendor. These details all together underscore the appropriateness for the term traditional to denote Factor 1.

The second pattern, Factor 2, which heavily features fruits and vegetables, plus smaller yet substantial quantities of legumes, legume products, and milk, was named a prudent dietary pattern, a term that other studies have extensively applied to reflect vegetable and fruit intake [19,20,34,38,39]. The prudent dietary pattern does not have any coefficients with negative values. The third pattern, Factor 3, has high loadings on cereals and cereal products, and a high negative loading was evident with rice, seafood, and vegetables; therefore, this pattern was labelled a modern dietary pattern. Because rice and fish were negatively loaded on Factor 3, but cereals and cereal products were loaded positively, this contrast was indicative of the shift from a traditional dietary pattern to a modernised dietary pattern, which entails a greater supply of refined wheat and grain. This shift has been extensively observed in many countries that undergo nutrition transition. The refined supply of wheat and grain has continued to increase gradually, but rice still dominates the dietary meal pattern in several transitioning countries [40]. Factor 4, the fourth pattern, which was dubbed a western dietary pattern, was characterised by high intake of meats, particularly processed meats, and low intake of non-alcoholic beverages; characteristic of the typical Western diet is high consumption of meats and processed meats. The fifth pattern, Factor 5, loaded highly on alcoholic beverages, eggs, and other meat, followed by non-alcoholic beverages, with negative loadings on confectioneries, and it was named a Chinese dietary pattern because it loaded highly on alcoholic beverages and other meat. These two foods are most commonly consumed by the Chinese population, and Malay respondents do not consume these two foods because of Islam’s prohibition of alcohol and certain meats. The sixth and final pattern, Factor 6, had high positive loadings on processed dairy products and spreads and was dubbed a combination dietary pattern because of its fusion of the elements of traditional and modern dietary patterns [34]. All in all, the patterns observed in this study provide an indication of the most common foods in respondents’ diets. 

Table 6 summarises the effects of the application of PCA to demographic groups evaluating which are distinguished by the selected pattern and reports food intakes (men, women, Malay, Chinese, Indian, and Other). All the six-food loads were categorised as gender (men, women) and ethnicity (Malay, Chinese, Indian, and Other). This was done in order to provide an outline of the characterization of the people of Malaysia by the chosen pattern. As can be seen in Table 6, men had a higher mean intake of factor loading in contrasts with women for PC1 (traditional dietary pattern), while Malay had a higher mean intake for PC1 compared to other ethnicities. In PC2 (prudent dietary pattern) women were observed to have greater intakes of fruit and milk while men had greater intakes of vegetables and legumes. Chinese was the predominant dietary pattern from PC2 (referring to highest intake for factor loadings of fruits and vegetables). Men and Indians have had a higher overall dietary intake of PC3 (modern dietary pattern). The negative PC3 loads were the least filled by the Indian. Men had higher consumption levels than women for PC4 (Western dietary pattern), while Malay had the largest intakes dependent on meat and refined meat loads. Males had a greater average intake of PC5 (Chinese dietary pattern) in comparison to women, compared to Chinese men, who had the highest intake among other ethnic groups with high alcoholic and other meat levels compared to other ethnic groups. The least of the Chinese was the negative loading of the confectionary. Men and Malay had higher intakes of processed dairy products for PC6 (combination dietary pattern’). While the Other ate refined milk products least. Overall, this result is capable of associating dietary habits with the demographic group analysed. The utility of this study depends on the identification of subjects that could be required for a nutrition policy intervention. Results can only be exploited if anyone can grasp who is correlated with dietary patterns. This allows the formulation of food-based dietary recommendations or planning other appropriations/incentives/duty-excises/subsidies or the development of a new food-based setting milieu.

### 3.4. Correlation among Selected Nutrient Intake, BMI and Composite Factor Scores

Once the dietary patterns were determined, scores for all respondents on each identified pattern were generated. These computed scores were used in determining correlation between BMI and other nutritional profiles (i.e., fat, added sugar, saturated fat, salt, and dietary fibre). Table 7 presents the correlation between six dietary patterns and BMI, total energy intake and intake of selected nutrients. It is apparent from the table that there were only a few variables that were not significantly correlated with one another at an α value of 0.05. However, the majority of the variables had weak correlation whilst a minority had moderate correlation. Almost all dietary patterns have a significant negligible BMI association, except for western and combination, which had no significant correlation. The traditional dietary pattern showed a moderate, positive correlation with total protein and total sugar intake. The sample correlation coefficients were 0.298 and 0.370, indicating that approximately 9% of the variance in the traditional dietary pattern can be explained by total protein and 14% of the variance in the traditional dietary pattern can be explained by total sugar, respectively. The correlation between this dietary pattern and protein may be due to high consumption of fish and seafood, which provide high-quality protein [35], in this diet. High consumption of confectioneries may be a plausible explanation for the moderate correlation between the traditional dietary pattern and total sugar intake. There was a significant moderate correlation between the prudent dietary pattern and dietary fibre: *r* = 0.482, *p* < 0.05. The sample correlation coefficient was 0.23, indicating that approximately 5% of the variance in the prudent diet can be explained by total dietary fibre intake. Meanwhile, the modern, western, and combination dietary patterns show a negligible significant relationship among the selected variables. There was a moderate positive correlation between the Chinese dietary pattern and total energy: *r* = 0.296, *p* < 0.05. The Chinese dietary pattern, which largely includes other meats (e.g., pork, ham, and bacon) and alcoholic beverages, may explain the increases in total protein and total energy, respectively. 

## 4. Discussion

In essence, the majority of the respondents belonged to at least one of the following demographics: residence in an urban area, female, Malay, married, employed, completed upper secondary education but not tertiary education and with a household income of less than MYR 1500, and comparison with the variance in other national sources suggested that the socio-demographic data in this study were representative of the Malaysian population. The average BMI in this study was 23.5, a value within the normal range. Total energy intake was highest among Malays and lowest among Indians. Both men and women had carbohydrate, protein and fat intake that exceeded the daily recommended allowances. The mean total carbohydrate intake was highest among Malay respondents and lowest among Chinese respondents. There was a significant difference in protein and fat intake among the Malay, Chinese, and Indian populations after adjustments for gender; however, intake of both nutrients demonstrated a minor effect. It is vital to understand the variations in nutrient intake among the different ethnic groups because this diversity reflects the cultural, religious, geographical, and genetic idiosyncrasies of each ethnicity [41].

On the basis of these dietary pattern findings, it is clear that Malaysia is undergoing a nutritional transition, in confirmation of national survey data. Dietary patterns have changed rapidly in Malaysia and in most other LMICs worldwide, and this transition has been marked by the Westernisation and modernisation of traditional diets, which greater consumption of meat, fat, salt, and sugar; dishes prepared outside the home; and pre-processed foods have supplanted [42,43]. The dietary pattern of Malaysia is generally more energy dense, with a high intake of sugar and fat, compared to the time trend in serial surveys [44,45], and the modern and western dietary patterns can reflect this. In most LMICs, dietary patterns shift mainly due to rapid changes in globalisation and urbanisation [46]. In recent literature, several mechanisms have been identified as contributing factors to nutrition transitions: worldwide food trade, foreign investment, global food advertising and promotion, supermarket development, emergence of global agri-business and transnational food companies, development of global food production rules, trade and distribution, urbanisation, and cultural change [47]. These factors have, in turn, triggered LMIC economic growth, and as an LMIC, Malaysia has recognised that these factors contribute to the nation’s economic growth. The dramatic spread of the fast food industry is one such example; the estimated total sales of this sector rose from RM 1 billion (US$263 million) in 1997 to RM 1.3 billion (US$340 million) in 2000 [44]. The increased availability of highly processed foods over time has influenced traditional cooking methods to favour “prepared to eat” or “minute” meals [48]. The emergence of the fast food and food service industries has paved the way for Malaysians to eat more food outside their homes (FAFH); nearly 11% of Malaysians bought food from dining establishments in 1999, up from 5% in 1973. The frequency of at-home dining, in contrast, fell from 34% in 1973 to 22% in 1999 [49]. FAFH consumption has been shown to be more energy dense and higher in total fat, saturated fat, and cholesterol but lower on a per-calorie basis in dietary fibre, calcium, and iron than food at home (FAH) [50,51,52,53]. In Malaysia, food services include road/hawker stalls, restaurants, and fast food outlets [54]. Furthermore, the proliferation of transnational supermarkets has improved the accessibility of affordable, imported, processed food [55], and the Malaysian government has amended the guidelines of the Foreign Investment Committee to expand flexibility for the participation of foreign equity in local companies [56]. These transnational supermarkets, especially in urban areas, have been widely accepted due to urbanisation and lifestyle changes, mainly in urban areas. Finally, this nutritional transition is marked by a shift from relatively monotonous diets of varying nutritional quality to a more varied and usually processed diet, which includes more food of animal origin, and more added sugar and fat [57].

Nearly all dietary patterns show a negligible significant correlation with BMI. This result was slightly different from those of other studies, which identified a good correlation between dietary pattern and obesity through factor analysis [21,58,59,60,61]. For instance, Dugee et al. (2009) identified three dietary patterns defined by PCA among Mongolians: (1) transitional high in processed meat and potatoes; (2) traditional rich in whole milk, fats, and oils; and (3) healthy, with greater intake of whole grains, mixed vegetables, and fruits [60]. It was found that the highest quintile in the transitional pattern was linked with a significantly greater risk of obesity, in contrast to the higher quintile for the healthy pattern, which was linked with significantly lower risk of obesity. Among the few studies on Asians populations in regard to dietary patterns and obesity, Kim et al. (2012) conducted a cross-sectional study among 10,089 Korean adults aged 19 years and older who had participated in the second and third Korean National Health and Nutrition Examination Surveys [61]. Upon performing PCA, four dietary patterns emerged: (1) the white rice and kimchi pattern; (2) the high-fat, sweets, and coffee pattern; (3) the meat and alcohol pattern; and (4) the grains, vegetables, and fish pattern. The Western dietary pattern, consisting of high fat consumption, sweets, and coffee, had a significant positive association with obesity after adjustments for socio-demographic and lifestyle factors. Therefore, this study concluded that this dietary pattern was associated with obesity among the Korean population [61]. Okubo et al. (2008) also conducted a cross-sectional study to link dietary patterns and obesity among the Japanese population. They applied PCA and discovered four dietary patterns among 3760 Japanese respondents: the healthy pattern, the Japanese traditional pattern, the Western pattern, and the coffee and dairy products pattern [59]. The findings showed that there were associations between dietary patterns and obesity among the Japanese population for the first three of these patterns; the healthy pattern, which was characterised by high intake of vegetables, mushrooms, seaweeds, potatoes, fish and shellfish, soy products, processed fish, fruit and salted vegetables, showed an inverse relationship with BMI. Meanwhile, the Japanese traditional pattern, which consisted of rice, miso soup and soy products, and the Western pattern, which comprised meats, fats and oils, seasonings, processed meats, and eggs, each had a positive relationship with BMI. In another study, the Japanese traditional pattern was also found to be significantly associated with impaired glucose tolerance (*p* for trend = 0.048) [62]. Sichieri (2002) brought attention to the association between dietary patterns and obesity among the Brazilian population [58] in a study that involved 2589 adults, aged 20 to 60, living in Rio de Janeiro. After performing PCA on the dietary intake of the population, three dietary patterns were derived: (1) the “mixed pattern”, which consisted of all food groups except rice and beans; (2) the “traditional pattern”, characterised by rice and beans; and (3) the “Western diet”, which comprised butter, margarine, and added sugar (e.g., in sodas). In this study, the traditional diet showed a significant negative association with excessive BMI after adjustment for age, leisure physical activity and occupation. Sherafat-Kazemzadeh et al.’s (2010) study in Iran on the association between dietary pattern and BMI is crucial for the Middle East as the region grapples with a high prevalence of obesity [63]. The authors conducted a prospective cohort study in Tehran, involving 141 subjects, for a six-year follow-up study [64]. The study performed RRR in deriving dietary patterns and revealed five such patterns: the traditional pattern, the fibre and PUFA pattern, the fibre and dairy pattern, the dairy pattern, and the egg pattern. The traditional pattern showed a positive association with obesity indices (BMI, WC, and WHR); it consisted of hydrogenated and saturated fat sources, eggs, red and processed meat, refined carbohydrates, vegetables, whole grain, and starchy vegetables. In light of Sherafat-Kazemzadeh et al.’s findings, it appears that only a certain type of dietary pattern is associated with obesity among the Iranian population [64]. A possible explanation for these results may be the heterogeneity of dietary intake patterns derived by factor analysis and lack of gold standards in applying the techniques [65]. Newby and Tucker (2004) seconded this hypothesis; they asserted that some studies showed no correlation between dietary patterns defined by factor analysis and diet-related diseases, and inconsistencies were obvious [11]. Inconsistencies may exist as a result of several methodological matters involved with factor analysis, which undergoes several decision-making processes that may affect the number and type of patterns that are obtained, stated, and evaluated [11]. In reference to the previous method discussed in factor analysis, the initial step in making decisions is either to group food items into smaller numbers or simply to analyse all food items for entry analysis. If the researcher chooses to group the food items then, again, the researcher must decide how to group them. The next decision relates to the number of patterns to retain in the final solution and then, finally, to labelling the patterns for easier identification. Newby and Tucker identified all these somewhat subjective decisions as bearing an impact on pattern analysis [11]. Investigation and improvement of dietary patterns in a multi-ethnic population is one of the most challenging tasks in the overall effort to straddle the fine line between over- and undernutrition. More perplexing is the analysis of the association between dietary patterns and obesity among multi-ethnic populations. In light of the few studies on the relationship between dietary patterns and obesity, this study endeavoured to discover the connection between Malaysian dietary patterns and obesity via PCA.

## 5. Conclusions

This study was designed to explore dietary pattern approaches by PCA approach and its relationship with nutrient intake and BMI. Some components derived from the factor loadings showed a positive correlation with several nutrient markers, namely,

(i) There was a moderate, positive correlation between the “traditional” dietary pattern and both total protein and total sugar intake;
(ii) There was a significant moderate correlation between the “prudent” dietary pattern and dietary fibre;
(iii) There was a moderate, positive correlation between the “Chinese” dietary pattern and total energy.

It is clear that further study of pattern analysis is warranted among the Malaysian population to produce validity and reproducibility for this dietary approach, especially due to the numerous methodological issues in running PCA. Only after developing a clear set of definitions for the patterns among the Malaysian population can researchers determine correlations between pattern analysis and disease outcomes in the future.

## Figures and Tables

**Table 1:** Frequencies and percentages for socio-demographic characteristics of the respondents (*n* = 3063).

	Frequency	Percent (%)
State of Malaysia		
Peninsular	2373	77.5
Southern	577	18.8
Central	987	32.2
Eastern	424	13.8
Northern	387	12.6
Sabah	360	11.8
Sarawak	330	10.8
Strataabl		
Urban	1590	51.9
Metropolitan city	1139	39.1
Big city	453	14.8
Rural	1473	48.1
Small town	380	12.4
Village	1093	35.9
Gender		
Male	1493	48.7
Female	1570	51.3
Ethnicity		
Malay	1602	52.3
Chinese	746	24.4
Indian/Punjabi	252	8.2
Other	463	15.4
Religion		
Muslim	1859	60.7
Buddhist	621	20.3
Christian	210	6.9
Hindu	308	10.1
Other	65	2.1
Marital Status		
Single	894	29.2
Married	2074	67.7
Divorced/Separated	36	1.2
Widow	56	1.8
Age (Mean Age)	34.5 ± 11.1 (SD)	
18–19	223	7.3
20–24	499	16.3
25–29	460	15.0
30–34	411	13.4
35–39	454	14.8
40–44	418	13.6
45–49	244	8.0
50–54	208	6.8
55–59	146	4.8
Level of Education		
Primary School	607	19.8
Lower Secondary School	642	21.0
Upper Secondary School	1076	35.1
Post-Secondary	176	5.7
College/University	436	14.2
Others	126	4.1
Employment Status		
Working	1968	64.2
Retired	28	9.2
Student	164	5.4
Housewife	735	24.0
Unemployed	108	3.5
Others/Refused to Answer	60	2.0
Monthly Household Income (Mean)	1992.61 ± 2752.8 (SD)	
<MYR 1500	1695	55.3
MYR 1500–3500	954	31.1
>MYR 3500	414	13.5
Body Mass Index (BMI)	23.5 ± 4.3 (SD)	
Underweight	333	10.9
Normal	1691	55.2
Overweight	806	26.3
Obese	233	7.6

MYR = Malaysian Ringgit (MYR) is the currency of Malaysia.

**Table 2:** Nutrient intake of men in a day by age groups and comparing nutrient intake with Recommended Nutrient Intake (RNI).

Dietary Intake	Mean ± S.E.				RNI	RNI Achievement
	18–29 Years (*n* = 580)	30–50 Years (*n* = 765)	51–60 Years (*n* = 151)	Total (*n* = 1496)		Mean% RNI
Energy Intake (kcal)	2463 ± 18.0	2491 ± 14.1	2408 ± 33.6	2472 ± 10.6	2440–2460	101
Carbohydrates (g)	461 ± 4.2	463 ± 3.3	446 ± 7.5	460 ± 2.5	NA ^b^ (55–70% of TE)	74.4 ^b^
Proteins (g)	110 ± 1.5	114 ± 1.5	106 ± 3.2	112 ± 1.0	62 g (15–20% of TE)	180.4
Fats (g)	65 ± 1.9	67 ± 2.1	58 ± 4.4	65 ± 1.4	54–82 g (20–30% of TE)	120.7
Calcium (mg)	788 ± 13.3	800 ± 11.7	782 ± 25.8	794 ± 8.3	1000	79.4
Iron ^a^ (mg)	19 ± 0.3	20 ± 0.2	19 ± 0.6	19 ± 0.2	14	138.9
Zinc (mg)	9 ± 0.3	9 ± 0.3	8 ± 0.7	9 ± 0.2	6.7	135.8
Thiamine (mg)	2 ± 0.0	2 ± 0.1	2 ± 0.1	2 ± 0.0	1.2	153.5
Riboflavin (mg)	2 ± 0.0	2 ± 0.0	2 ± 0.1	2 ± 0.0	1.3	170.9
Niacin (mg NE)	19 ± 0.6	20 ± 0.6	17 ± 1.2	19 ± 0.4	16	120.2
Vitamin C (mg)	120 ± 3.3	129 ± 3.3	132 ± 8.3	126 ± 2.3	70	179.9
Vitamin A (µg)	580 ± 152.1	631 ± 130.7 *	653 ± 378.6	614 ± 97.2	600	102.3
Vitamin E (mg)	12 ± 0.4	14 ± 0.4 *	13 ± 0.9	13 ± 0.3	10	103.2
Selenium (µg)	44 ± 1.0	40 ± 0.8 *	36 ± 1.8 *	41 ± 0.6	33	124.3

^a^ The recommended iron is based on a 10% iron bioavailability level. ^b^ NA; recommendation is based on percentage from total energy intake (TE). * Significantly different from age group 18–29 years old, *p* < 0.05 (one-way ANOVA with Bonferroni correction). RNI = Recommended Nutrient Intakes for Malaysia.

**Table 3:** Nutrient intake of women in a day by age groups and comparing nutrient intake with Recommended Nutrient Intake (RNI).

Dietary Intake	Mean ± S.E.				RNI	RNI Achievement
	18–29 Years (*n* = 607)	30–50 Years (*n* = 814)	51–60 Years (*n* = 161)	Total (*n* = 1582)		Mean% RNI
Energy intake (kcal)	2083 ± 14.9	2159 ± 13.3 *	2112 ± 28.1	2125 ± 9.4	2000–2180	100.8
Carbohydrates (g)	393 ± 3.5	403 ± 3.1	391 ± 7.2	398 ± 2.2	NA ^b^ (55–70% of TE)	74.9 ^b^
Proteins (g)	91 ± 1.1	97 ± 1.2 *	91 ± 1.9	94 ± 0.8	55 (15–20% of TE)	171.2
Fats (g)	52 ± 1.4	53 ± 1.5	42 ± 1.5 *	51 ± 0.9	46–70 (20–30% of TE)	111.8
Calcium (mg)	705 ± 11.0	723 ± 10.0 **	659 ± 22.9	709 ± 7.1	800 (18–50 years) 1000 (51–60 years)	87.0
Iron ^a^ (mg)	17 ± 0.2	18 ± 0.2 *	17 ± 0.4	17 ± 0.1	29 (18–50 years) 11 (51–60 years)	69.0
Zinc (mg)	7 ± 0.2	7 ± 0.2	5 ± 0.2	7 ± 0.2	4.9	143.7
Thiamine (mg)	1 ± 0.0	2 ± 0.0 **	1 ± 0.0	1 ± 0.0	1.1	133.5
Riboflavin (mg)	2 ± 0.0	2 ± 0.0	2 ± 0.0	2 ± 0.0	1.1	174.7
Niacin (mg NE)	15 ± 0.4	16 ± 0.4	14 ± 0.6	15 ± 0.3	14	110.4
Vitamin C (mg)	126 ± 3.5	128 ± 3.1	118 ± 7.3	126 ± 2.2	70	180.5
Vitamin A (µg)	552 ± 138.4	601 ± 123.8	570 ± 275.6	579 ± 87.7	500	115.9
Vitamin E (mg)	11 ± 0.3	12 ± 0.3 *	10 ± 0.5	11 ± 0.2	7.5	105.0
Selenium (µg)	36 ± 0.8 **	35 ± 0.6 **	33 ± 1.7	35 ± 0.5	25	140.5

^a^ The recommended iron is based on a 10% iron bioavailability level. ^b^ NA; recommendation is based on percentage from total energy intake (TE). * Significantly different from age group 18–29 years old, *p* < 0.05 (one-way ANOVA with Bonferroni correction). ** Significantly different from age group 51–60 years old, *p* < 0.05 (one-way ANOVA with Bonferroni correction). RNI = Recommended Nutrient Intakes for Malaysia.

**Table 4:** Distribution of nutrient intake according to ethnicity in Malaysia.

Dietary Intake	Mean ± S.E.			
	Malay	Chinese	Indian	Other
Energy Intake (kcal)	2307 ± 10.6	2287 ± 15.8	2254 ± 28	2281 ± 20.1
Carbohydrates (g)	430 ± 2.4	428 ± 3.5	433 ± 6.1	419 ± 4.4
Proteins (g)	106 ± 0.9	99 ± 1.1	88 ± 2.0	105 ± 1.8
Fats (g)	61 ± 1.2	53 ± 1.5	54 ± 2.5	60 ± 2.5
Calcium (mg)	786 ± 8.0	696 ± 9.7	741 ± 18.7	718 ± 13.7
Iron ^a^ (mg)	19 ± 0.2	18 ± 0.2	16 ± 0.3	19 ± 0.3
Zinc (mg)	9 ± 0.2	7 ± 0.2	6 ± 0.4	9 ± 0.4
Thiamine (mg)	2 ± 0.03	2 ± 0.03	2 ± 0.06	2 ± 0.06
Riboflavin (mg)	2 ± 0.8	2 ± 0.7	2 ± 0.7	2 ± 0.8
Niacin (mg NE)	18 ± 0.4	17 ± 0.4	16 ± 0.7	18 ± 0.7
Vitamin C (mg)	126 ± 2.2	128 ± 3.2	120 ± 5.2	127 ± 4.0
Vitamin A (µg)	568 ± 86.0	633 ± 133.2	468 ± 159.1	698 ± 198.0
Vitamin E (mg)	14 ± 0.3	10 ± 0.3	9 ± 0.5	14.0 ± 11.7
Selenium (µg)	38 ± 0.5	40.0 ± 0.7	37 ± 1.3	35 ± 1.0

^a^ The recommended iron is based on a 10% iron bioavailability level.

**Table 5:** Factor loading matrix for the six dietary patterns identified.

Principal Components	Positive Scoring Coefficients	Negative Scoring Coefficients	Variance Explained (%)
Principal Component 1 (PC 1)	Flavourings (0.62)	Other meat (−0.45)	9.9
“traditional”	Fish and seafood (0.56)	Milk (−0.23)	
	Confectionery (0.55)	Cereals and cereal products (−0.22)	
Principal Component 2 (PC 2)	Fruits (0.65)		9.5
“prudent”	Vegetables (0.64)		
	Legumes and legume products (0.53)		
	Milk (0.34)		
Principal Component 3 (PC 3)	Cereals and cereal products (0.68)	Rice (−0.70)	7.3
“modern”		Fish and seafood (−0.26)	
		Vegetables (−0.25)	
Principal Component 4 (PC 4)	Meats (0.68)	Non-alcoholic beverages (−0.29)	7.0
“western”	Processed meats (0.67)		
Principal Component 5 (PC 5)	Alcoholic beverages (0.6)	Confectionery (−0.24)	6.4
“Chinese”	Eggs (0.43)		
	Other meat (0.46)		
	Non-alcoholic beverages (0.34)		
Principal Component 6 (PC 6)	Processed dairy products (0.8)		5.9
“combination”	Spreads (0.5)		

Extraction method: Principal Component Analysis. Rotation method: Varimax with Kaiser Normalisation. Rotation converged in 9 iterations. Loadings ≥0.2 were only selected. Note: Each item has a high, or meaningful, loading on one factor only even though factor loading |*r*| ≥ 0.2 [30].

**Table 6:** Mean grams food consumed by food groups of derived dietary pattern and the analysed population groups.

PC. Food Group (Overall Factor Loading)	Men (*n* = 1493)	Women (*n* = 1570)	Malay (*n* = 1602)	Chinese (*n* = 746)	Indian (*n* = 252)	Other (*n* = 463)
	Mean Intake (gram) ± Standard Error					
PC 1. Flavourings (0.62)	40.23 ± 0.90	35.00 ± 0.76	45.71 ± 0.86	25.43 ± 0.96	26.76 ± 1.53	34.73 ± 1.42
PC 1. Fish and seafood (0.56)	119.12 ± 2.19	109.14 ± 1.98	134.24 ± 2.05	78.95 ± 2.26	68.07 ± 3.58	125.48 ± 4.25
PC 1. Confectionery (0.55)	81.88 ± 1.60	80.21 ± 1.50	90.57 ± 1.54	56.64 ± 1.68	68.62 ± 2.89	94.01 ± 3.34
PC 1. Other meat (−0.45)	9.78 ± 0.68	7.52 ± 0.50	0.02 ± 0.02	29.49 ± 1.33	0.49 ± 0.19	9.19 ± 0.95
PC1. Milk (−0.23)	7.49 ± 0.73	10.49 ± 0.88	7.71 ± 0.67	10.90 ± 1.71	13.46 ± 1.41	8.16 ± 0.93
PC 1. Cereals and cereal products (−0.22)	262.87 ± 3.37	207.42 ± 2.74	220.51 ± 2.72	269.10 ± 5.00	291.35 ± 8.45	195.86 ± 5.33
PC 2. Fruits (0.65)	197.69 ± 4.11	207.55 ± 4.11	199.13 ± 4.11	217.01 ± 5.87	203.89 ± 9.72	191.65 ± 7.04
PC 2. Vegetables (0.64)	136.60 ± 2.95	132.09 ± 2.60	124.05 ± 2.69	154.95 ± 4.09	104.89 ± 4.90	152.44 ± 5.25
PC 2. Legumes and legume products (0.53)	27.23 ± 0.82	25.34 ± 0.73	26.32 ± 0.80	28.85 ± 1.10	29.36 ± 1.82	20.16 ± 1.05
PC 2. Milk (0.34)	7.49 ± 0.73	10.49 ± 0.88	7.71 ± 0.67	10.90 ± 1.71	13.46 ± 1.41	8.16 ± 0.93
PC3. Cereals and cereal products (0.68)	262.87 ± 3.37	207.42 ± 2.74	220.51 ± 2.72	269.10 ± 5.00	291.35 ± 8.45	195.86 ± 5.33
PC3. Rice (−0.70)	363.09 ± 4.46	307.08 ± 3.61	324.93 ± 3.86	322.63 ± 5.19	285.13 ± 7.83	412.81 ± 9.21
PC3. Fish and seafood (−0.26)	119.12 ± 2.19	109.14 ± 1.98	134.24 ± 2.05	78.95 ± 2.26	68.07 ± 3.58	125.48 ± 4.25
PC3. Vegetables (−0.25)	136.60 ± 2.95	132.09 ± 2.60	124.05 ± 2.69	154.95 ± 4.09	104.89 ± 4.90	152.44 ± 5.25
PC4. Meats (0.68)	46.19 ± 1.22	33.34 ± 0.88	41.86 ± 1.06	40.70 ± 1.56	28.08 ± 1.99	36.31 ± 1.91
PC4. Processed meats (0.67)	11.49 ± 0.42	10.08 ± 0.33	12.32 ± 0.41	8.08 ± 0.41	9.06 ± 0.76	10.65 ± 0.64
PC4. Non-alcoholic beverages (−0.29)	2215.43 ± 26.13	1920.05 ± 19.45	2045.16 ± 22.20	1992.08 ± 34.32	2178.98 ± 54.98	2182.66 ± 42.97
PC5. Alcoholic beverages (0.6)	5.00 ± 0.92	1.10 ± 0.18	0.05 ± 0.03	8.98 ± 1.71	4.13 ± 1.06	2.97 ± 1.01
PC5. Eggs (0.43)	34.93 ± 0.81	26.83 ± 0.65	32.38 ± 0.72	29.99 ± 1.00	21.32 ± 1.31	31.65 ± 1.55
PC5. Other meat (0.46)	9.78 ± 0.68	7.52 ± 0.50	0.02 ± 0.02	29.49 ± 1.33	0.49 ± 0.19	9.19 ± 0.95
PC5. Non-alcoholic beverages (0.34)	2215.43 ± 26.13	1920.05 ± 19.45	2045.16 ± 22.20	1992.08 ± 34.32	2178.98 ± 54.98	2182.66 ± 42.97
PC5. Confectionery (−0.24)	81.88 ± 1.61	80.21 ± 1.50	90.57 ± 1.54	56.64 ± 1.68	68.62 ± 2.89	94.01 ± 3.34
PC6. Processed dairy products (0.8)	36.26 ± 1.09	22.80 ± 0.80	36.25 ± 1.06	20.48 ± 1.06	30.13 ± 2.48	19.41 ± 1.26
PC6. Spreads (0.5)	7.09 ± 0.29	7.16 ± 0.25	6.79 ± 0.27	8.25 ± 0.42	8.38 ± 0.75	5.80 ± 0.37

PC 1 = Principal component 1 referring to “traditional” dietary pattern. PC 2 = Principal component 2 referring to “prudent” dietary pattern. PC 3 = Principal component 3 referring to “modern” dietary pattern. PC 4 = Principal component 4 referring to “western” dietary pattern. PC 5 = Principal component 5 referring to “Chinese” dietary pattern. PC 6 = Principal component 6 referring to “combination” dietary pattern. Malay, Chinse, and Indians are the major ethnicity in Malaysia, while other refers to Bumiputra Sabah and Sarawak as well as indigenous people.

**Table 7:** Correlation coefficients among six food patterns and total energy intake, nutrient intake and BMI of respondents.

Energy and Nutrients	Factor 1 “Traditional”	Factor 2 “Prudent”	Factor 3 “Modern”	Factor 4 “Western”	Factor 5 “Chinese”	Factor 6 “Combination”
BMI	0.148 *	0.121 *	0.079 *	−0.024	0.080 *	−0.012
Total Energy (kcal)	0.254 *	0.187 *	0.158 *	0.183 *	0.296 *	0.120 *
Total Carbohydrate (g)	0.142 *	0.145 *	0.199 *	0.031	0.167 *	0.055 *
Total Protein (g)	0.298 *	0.275 *	0.001	0.188 *	0.259 *	0.046 *
Total Fat (g)	0.145 *	0.277 *	0.092 *	0.096 *	0.087 *	0.093 *
Total Dietary Fibre (g)	0.081 *	0.482 *	0.043	0.011	0.051 *	−0.011
Total Sugar (g)	0.370 *	0.249 *	0.112 *	0.066 *	−0.036 *	0.098 *
Total Saturated Fat (g)	0.109 *	0.247 *	0.044 *	0.213 *	0.146 *	0.270 *
Total Sodium (mg)	0.455	0.115 *	−0.019	0.195 *	0.149 *	0.039 *

* Correlation is significant at the 0.05 level (2-tailed). BMI = body mass index.

## Data Availability

Restrictions apply to the availability of these data. Data was obtained from Institute of Public Health, Malaysia (IPH) and are available from the authors with the permission of IPH.

## Appendix A

**Table A1:** List of 126 food items grouped into 17 food item groups.

No.	Original Food Items	New Food Groups	No.	Original Food Items	New Food Groups	No.	Original Food Items	New Food Groups
1	Rice	Rice	22	Meat Burger	Processed meats	43	Duck Egg	Eggs
2	Rice Porridge	Rice	23	Hot Dog	Processed meats	44	Quail Egg	Eggs
3	Glutinous Rice	Cereals and cereals products	24	Nugget	Processed meats	45	Salted Egg	Eggs
4	Noodles	Cereals and cereals products	25	Chicken Ball	Processed meats	46	Pulses	Legumes and legume products
5	Vermicelli	Cereals and cereals products	26	Ham	Other meat	47	Soybean	Legumes and legume products
6	Lohshifun	Cereals and cereals products	27	Bacon	Other meat	48	Fermented Soy	Legumes and legume products
7	Pasta	Cereals and cereals products	28	Luncheon	Other meat	49	Groundnut	Legumes and legume products
8	Sagu	Cereals and cereals products	29	Pork	Other meat	50	Milk	Milk
9	Bread	Cereals and cereals products	30	Fish	Fish and seafood	51	Powdered Milk	Milk
10	Bun	Cereals and cereals products	31	Fresh Fish	Fish and seafood	52	Condensed Milk	Processed dairy products
11	Roti Canai	Cereals and cereals products	32	Anchovy	Fish and seafood	53	Evaporated Milk	Processed dairy products
12	Capati	Cereals and cereals products	33	Canned Fish	Fish and seafood	54	Flavoured Yogurt	Processed dairy products
13	Dosai	Cereals and cereals products	34	Cockles	Fish and seafood	55	Cheese	Processed dairy products
14	Cereals	Cereals and cereals products	35	Prawn	Fish and seafood	56	Leafy Vegetable	Vegetables
15	Ready-to-eat Cereals	Cereals and cereals products	36	Cuttlefish	Fish and seafood	57	Non-leafy Vegetable	Vegetables
16	Pizza	Cereals and cereals products	37	Dried Cuttlefish	Fish and seafood	58	Root	Vegetables
17	Corn	Cereals and cereals products	38	Crab	Fish and seafood	59	Cabbage	Vegetables
18	Chicken	Meat	39	Salted Fish	Fish and seafood	60	Pumpkin	Vegetables
19	Meat	Meat	40	Fish Balls	Fish and seafood	61	Salted Vegetables	Vegetables
20	Goat	Meat	41	Lekor	Fish and seafood	62	Ulam	Vegetables
21	Duck	Meat	42	Chicken Egg	Eggs	63	Baby Corn	Vegetables

**Table A2:** List of 126 food items grouped into 17 food item groups.

No.	Original Food Items	New Food Groups	No.	Original Food Items	New Food Groups	No.	Original Food Items	New Food Groups
64	Mushrooms	Vegetables	85	Dried Fruits	Fruits	106	Ice Cream	Confections
65	Sprouts	Vegetables	86	Plain Water	Non-alcoholic beverages	107	ABC	Confections
66	Papaya	Fruits	87	Tea	Non-alcoholic beverages	108	Jelly/Custard	Confections
67	Guava	Fruits	88	Coffee	Non-alcoholic beverages	109	Snacks	Confections
68	Mandarin Orange	Fruits	89	Chocolate Drink	Non-alcoholic beverages	110	Jam	Spreads
69	Mango	Fruits	90	Malted Drink	Non-alcoholic beverages	111	Seri Kaya	Spreads
70	Pineapple	Fruits	91	Rose Syrup	Non-alcoholic beverages	112	Butter	Spreads
71	Banana	Fruits	92	Fruit Juice	Non-alcoholic beverages	113	Margarine	Spreads
72	Watermelon	Fruits	93	Carbonated Drink	Non-alcoholic beverages	114	Peanut	Spreads
73	Starfruit	Fruits	94	Botanical Herbs	Non-alcoholic beverages	115	Cream Cheese	Spreads
74	Jackfruit	Fruits	95	Energy Drink	Non-alcoholic beverages	116	Sugar	Flavorings
75	Orange	Fruits	96	Soybean Drink	Non-alcoholic beverages	117	Honey	Flavorings
76	Apple	Fruits	97	Syandi	Alcoholic beverages	118	Shrimp Sauce	Flavorings
77	Pear	Fruits	98	Beer	Alcoholic beverages	119	Anchovy Sauce	Flavorings
78	Grape	Fruits	99	Wine	Alcoholic beverages	120	Shrimp Cencalok	Flavorings
79	Durian	Fruits	100	Spirit	Alcoholic beverages	121	Thick Soy Sauce	Flavorings
80	Rambutan	Fruits	101	Liquor	Alcoholic beverages	122	Light Soy Sauce	Flavorings
81	Longan Segar	Fruits	102	Local Delicacies	Confections	123	Ketchup Sauce	Flavorings
82	Laici Segar	Fruits	103	Cake	Confections	124	Oyster Sauce	Flavorings
83	Honeydew	Fruits	104	Biscuits	Confections	125	Fish Sauce	Flavorings
84	Tinned Fruits	Fruits	105	Sweets	Confections	126	Prawn Paste	Flavorings
